# Aerobic exercise partially improves the skeletal muscle phenotype in a model of heart failure with preserved ejection fraction in male mice

**DOI:** 10.14814/phy2.70962

**Published:** 2026-06-04

**Authors:** Cielo Martínez Martínez, Jorge Fragoso Medina, Estefanía Bravo Sánchez, Bianca Nieblas, Alfredo Saavedra Molina, Christian Cortés Rojo, Salvador Manzo Ávalos, Noemí García, Rocío Montoya Pérez

**Affiliations:** ^1^ Institute of Chemical‐Biological Research Michoacan University of Saint Nicholas of Hidalgo Morelia Michoacán México; ^2^ Institute for Obesity Research Tecnológico de Monterrey Monterrey Nuevo León México

**Keywords:** calcium, cardiovascular disease, muscle fatigue, oxidative stress, physical activity, skeletal muscle

## Abstract

Heart failure with preserved ejection fraction (HFpEF) is associated with skeletal muscle dysfunction that contributes to exercise intolerance and disease progression. Altered Ca^2+^ homeostasis and increased oxidative stress have been proposed as mechanisms underlying muscle weakness in HFpEF; however, the effects of aerobic exercise on skeletal muscle physiology in this condition remain unclear. This study evaluated the impact of moderate‐intensity aerobic exercise on skeletal muscle contractile function, fatigue resistance, oxidative stress, and mRNA expression of key Ca^2+^‐handling proteins in a model with features consistent with HFpEF. Male C57BL/6J mice were assigned to Control, Exercise, HFpEF (HFD + L‐NAME‐treated), and HFpEF+Exercise groups. HFpEF‐like features were induced by an 8‐week high‐fat diet combined with L‐NAME administration. During the final 4 weeks, mice underwent aerobic exercise training. Soleus muscles were analyzed using in vitro contractile recordings, oxidative stress markers, and RT‐qPCR for RyR1, SERCA1, and NCX3 mRNA expression. HFpEF (HFD + L‐NAME‐treated mice) showed reduced contractile force and fatigue resistance, increased oxidative stress, and decreased SERCA1 and NCX3 expression. Aerobic exercise partially restored muscle performance and improved redox balance without normalizing Ca^2+^‐handling gene expression. These findings suggest that aerobic exercise enhances skeletal muscle function in HFpEF‐like models, primarily by modulating redox homeostasis.

## 1 INTRODUCTION

Heart failure with preserved ejection fraction (HFpEF) is not merely a disease characterized by impaired cardiac relaxation, but a complex clinical syndrome affecting multiple physiological systems, including skeletal muscle (Adams et al., 2017; Gevaert et al., 2022). Skeletal muscle dysfunction in HFpEF is associated with reduced muscle strength and early fatigue. It represents a significant determinant of exercise intolerance, a key predictor of clinical outcomes in this condition (Gupte & Hamilton, 2016; Kelley et al., 2022; Saw et al., 2021). Consequently, improving functional capacity has become a therapeutic priority (Kittleson et al., 2023; Tucker et al., 2018).

Exercise tolerance largely depends on skeletal muscle metabolism, as this tissue accounts for the majority of oxygen consumption during physical activity (Paneroni et al., 2018). Efficient muscle performance requires tightly regulated Ca^2+^ signaling and a balanced redox environment involving reactive oxygen and nitrogen species (ROS/RNS), both of which are essential for excitation–contraction coupling (ECC) (Bolaños & Calderón, 2022).

In skeletal muscle, ECC is initiated by depolarization of the transverse tubules, which allows dihydropyridine receptors (DHPRs) to sense changes in membrane potential. DHPRs are mechanically coupled to ryanodine receptor type 1 (RyR1) in the sarcoplasmic reticulum (SR), triggering Ca^2+^ release into the sarcoplasm and initiating contraction. Muscle relaxation requires Ca^2+^ reuptake into the SR via the sarcoplasmic reticulum Ca^2+^‐ATPase 1 (SERCA1) and Ca^2+^ extrusion through the sodium/calcium exchanger type 3 (NCX3) at the sarcolemma (Bolaños & Calderón, 2022; Michelucci et al., 2021).

Alterations in ECC, including impaired Ca^2+^ release and reuptake as well as redox imbalance, have been reported in several muscle pathologies (Eshima, 2021; Michelucci et al., 2021; Qaisar et al., 2018); however, their contribution to skeletal muscle dysfunction in HFpEF remains poorly characterized. It has been proposed that increased oxidative stress and altered expression of genes regulating Ca^2+^ handling may contribute to muscle dysfunction and exercise intolerance in HFpEF (Eshima, 2021; Michelucci et al., 2021).

Paradoxically, moderate‐intensity aerobic exercise is a safe and effective therapeutic strategy for HFpEF, reducing associated comorbidities such as hypertension, type 2 diabetes mellitus, and obesity (Liang et al., 2021). In skeletal muscle, aerobic exercise prevents atrophy, improves strength, and attenuates oxidative stress across diverse pathological conditions (Gallagher et al., 2023; Margaritelis et al., 2020). Additionally, aerobic training has been shown to modulate the expression and activity of RyR1, SERCA1, and NCX3, thereby enhancing Ca^2+^ regulation and contractile performance (Bueno Jr et al., 2010; Ferreira et al., 2010).

In animal models of HFpEF, exercise improves skeletal muscle metabolism and exercise tolerance in mice with HFpEF induced by a high‐fat diet (HFD) and L‐NAME (Quiriarte et al., 2024). It attenuates functional impairments in the diaphragm and soleus of salt‐sensitive female Dahl rats with HFpEF (Bowen et al., 2015). These findings support a beneficial role of exercise on skeletal muscle across different HFpEF models.

However, the combined effects of aerobic exercise on skeletal muscle contractile function, oxidative stress, and Ca^2+^‐handling gene expression have not been systematically examined in HFpEF. In the present study, we used a previously validated mouse model that has been reported to exhibit features consistent with HFpEF induced by high‐fat diet and L‐NAME administration (Méndez‐Fernández et al., 2024; Schiattarella et al., 2019), which has been shown to reproduce several characteristics of the human disease, including preserved left ventricular ejection fraction, concentric hypertrophy, and exercise intolerance after 8 weeks of induction. We aimed to evaluate the impact of aerobic exercise on contractile force, oxidative stress, and mRNA expression of RyR1, SERCA1, and NCX3 in the soleus muscle, hypothesizing that aerobic exercise would improve skeletal muscle function, with potential involvement of mechanisms related to redox homeostasis and Ca^2+^‐handling gene expression.

## 2 MATERIALS AND METHODS

### 2.1 Ethical approval

The animal protocols were approved by the Bioethics and Safety Committee of the Instituto de Investigaciones Químico Biológicas at UMSNH (2022‐06). These procedures were conducted in accordance with the recommendations of the Mexican Official Standard NOM062‐ZOO‐1999, which establishes technical specifications for the care and use of laboratory animals. The animals were housed in acrylic cages within an animal facility with a 12‐h light–dark cycle and had free access to water and standard rodent chow (Rodent Diet 5001, SA de CV, México). Every effort was made to minimize animal pain and suffering at all times.

### 2.2 Preclinical model

**FIGURE 1:**
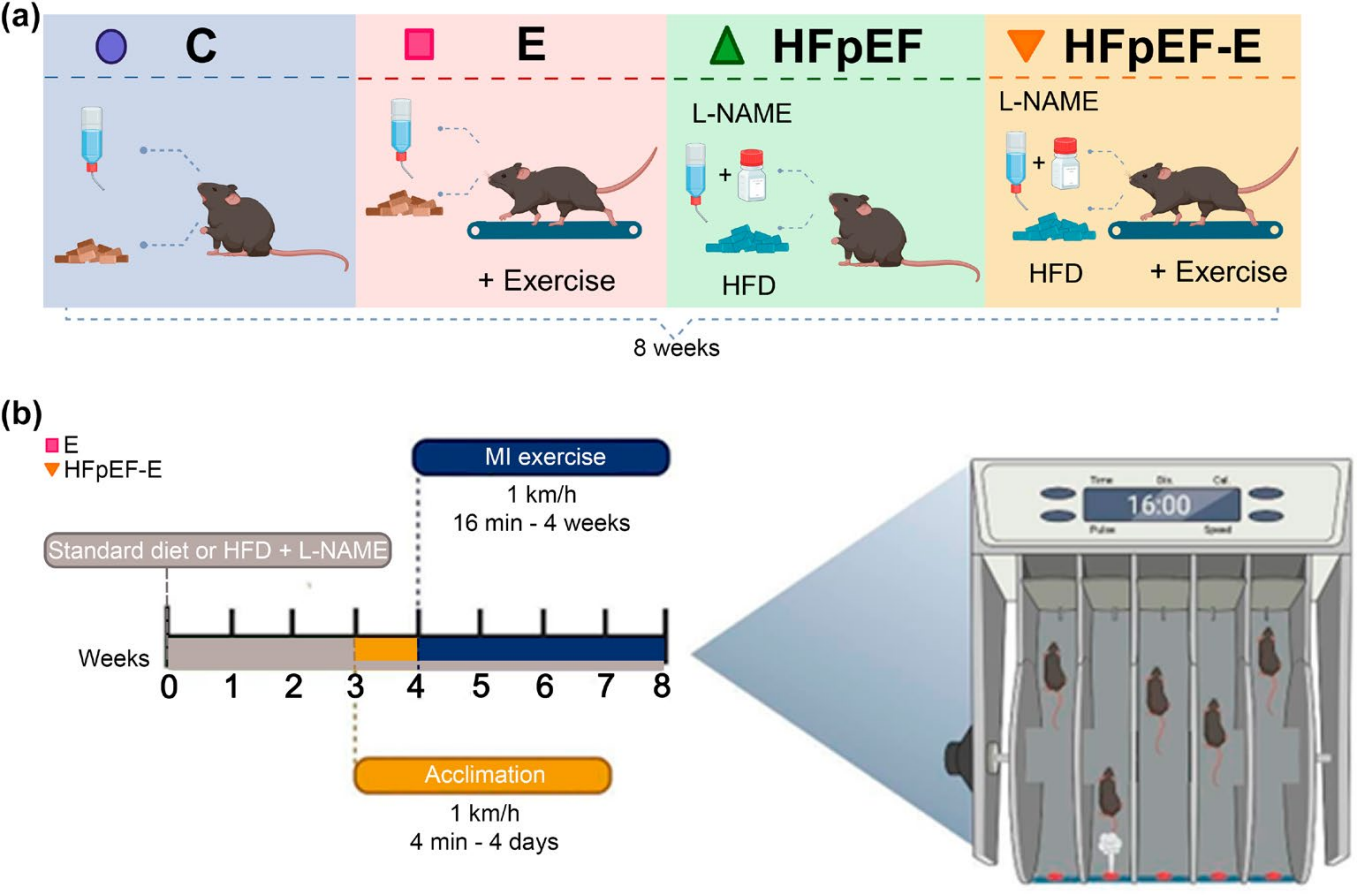
Experimental design. (a) Schematic representation of the dietary and exercise interventions applied to each mouse group. Control group (C): Standard diet and drinking water ad libitum. Exercise group (E): Standard diet, drinking water ad libitum, and moderate‐intensity aerobic exercise. Sedentary HFpEF group (HFpEF): High‐fat diet (60% lipids) and L‐NAME (0.5 g/L) in drinking water ad libitum. Exercised HFpEF group (HFpEF‐E): High‐fat diet (60% lipids) and L‐NAME (0.5 g/L) in drinking water ad libitum, plus moderate‐intensity aerobic exercise. (b) Timeline of the moderate‐intensity aerobic exercise protocol. All animals received continuous dietary and pharmacological treatment (standard diet or HFD + L‐NAME) throughout the 8‐week protocol (gray bar). Treadmill acclimation was performed during week 3 (1km/h, 4 min/day, 4 days). From weeks 4 to 8, animals underwent moderate‐intensity aerobic exercise (1 km/h, 16 min/session, 4 sessions/week). The treadmill used had individual lanes and an air‐puff stimulus to encourage running. E, exercise group; HFD, high‐fat diet; HFpEF‐E, HFpEF group with exercise; L‐NAME, Nω‐Nitro‐L‐arginine methyl ester; MI, moderate‐intensity. Created with BioRender.com.

HFpEF was induced in 8‐week‐old male C57BL/6J mice by administering a high‐fat diet (HFD; 58Y1‐DIO Rodent Purified Diet with 60% of energy derived from fat/Blue, TestDiet) in combination with 0.5 g/L Nω‐Nitro‐L‐arginine methyl ester (L‐NAME, N5751, Sigma‐Aldrich, St. Louis, MO, USA) in drinking water ad libitum for 8 weeks (Schiattarella et al., 2019). Control mice received a standard diet and ad libitum access to drinking water during the same period. Four experimental groups were established according to condition (HFpEF or control) and physical activity (sedentary or exercised) (Figure 1a).

This mouse model has been previously reported to reproduce key features of HFpEF, including preserved ejection fraction, concentric ventricular hypertrophy, pulmonary congestion, and exercise intolerance (Méndez‐Fernández et al., 2024; Quiriarte et al., 2024; Schiattarella et al., 2019). In the present study, because the primary objective was to evaluate skeletal muscle parameters, direct cardiac functional measurements (e.g., echocardiographic assessment of LVEF or E/E′ ratio) were not performed. Therefore, rather than assuming HFpEF, the findings are interpreted within the context of a model exhibiting features consistent with an HFpEF‐like phenotype, supported by prior validation and the phenotypic characteristics observed in this study.

### 2.3 Metabolic biomarkers

Weight and basal glucose were monitored in the groups after 12 h fasting using a scale and an Accu‐Chek® Performa glucometer (Roche Diagnostics, Mannheim, Germany), respectively, once a week at 8:00 am during the experimental protocol.

### 2.4 Moderate‐intensity aerobic exercise protocol

Mice in the E and HFpEF‐E groups were acclimated on a specialized treadmill equipped with grids that generate air puffs to stimulate movement. Acclimation was performed at a speed of 1 km/h and 0° incline for 4 min over 4 consecutive days. Subsequently, a moderate‐intensity aerobic exercise protocol was applied, defined as 70% of maximal running capacity in mice, based on previous reports with slight modifications (Martinez‐Huenchullan et al., 2018). The exercise protocol consisted of running sessions at 1 km/h with 0° incline, lasting 16 min per session, 4 times per week for 4 weeks (Figure 1b). All exercise sessions were conducted during the dark phase of the light/dark cycle in the animal facility to respect the mice's natural nocturnal activity and minimize stress.

### 2.5 Exercise exhaustion test

At the end of the treatment period, all experimental groups underwent an exercise exhaustion test. The exhaustion test was performed 48 h after the experimental groups completed their respective treatments. Sedentary groups were previously acclimated to the treadmill for 4 days, running at 1 km/h with a 0° incline for 4 min. The test began with a warmup phase at 1 km/h and 0° incline for 4 min. Afterward, the speed was increased by 0.1 km/h every 2 min until the mice reached exhaustion. Exhaustion was defined as the inability of the mouse to resume running within 10 s after direct contact with an air‐puff stimulus grid. During the test, the total running time (s) was recorded, and the running distance was calculated using the formula d = v × t (Davidson et al., 2006; la Fuente et al., 2019; Pareja‐Galeano et al., 2012).

### 2.6 Dissection and preservation of muscle and other tissues

Mice were sacrificed by cervical dislocation, a method selected to avoid the use of anesthetics or pharmacological agents that could alter the contractile properties of the soleus muscle during in vitro tension recordings. Death was confirmed visually and by the absence of corneal and limb reflexes, clearly indicating that the animals were deceased immediately after dislocation. A secondary invasive method was not required, in accordance with the AVMA *Guidelines for the Euthanasia of Animals* (2020) (Leary, 2020) and the protocol approved by the institutional ethics committee. All animals were sacrificed 48 h after completing the exercise exhaustion test to minimize the acute effects of the final exercise bout. Soleus muscles from both hind limbs were dissected and weighed for analysis. One soleus muscle was placed in a mouse‐formulated physiological solution (NaCl 148 mM, KCl 4.5 mM, CaCl_2_ 2 mM, MgCl_2_ 1 mM, NaHCO_3_ 12 mM, NaH_2_PO_4_ 0.44 mM, C_6_H_12_O_6_ 5.5 mM, pH 7.4) with continuous carbogen gas supply (95% O_2_, 5% CO_2_) for in vitro tension recording. Subsequently, it was preserved in a mouse‐formulated physiological solution and stored at −80°C. After preservation, the soleus muscle was homogenized, and its protein content was determined using the Lowry method (Lowry et al., 1951) with bovine serum albumin as the standard to perform the corresponding biochemical analyses.

The contralateral soleus muscle was preserved in RNAlater, kept at −4°C for 24 h, and then stored at −80°C for long‐term RT‐qPCR assays. Additionally, the heart and lungs were collected and weighed as indicators of cardiac hypertrophy and pulmonary congestion, respectively. The apex of the heart was fixed in 10% formaldehyde for subsequent histopathological analyses.

### 2.7 Cardiac histopathological analysis

For histopathological analysis, transverse heart sections at the level of the papillary muscles were obtained and stained with hematoxylin and eosin (H&E). Images were captured by digital photomicrography using an optical microscope (Leica DM3000, Leica Microsystems, Wetzlar, Germany) equipped with a 40× objective lens. Quantitative analysis of cardiomyocyte cross‐sectional area was performed by manually tracing cell borders in ImageJ (version 1.54g; National Institutes of Health, USA) to assess changes in cell size associated with cardiac hypertrophy. For each animal, at least 25 cardiomyocytes with a well‐defined central nucleus and clearly distinguishable borders were selected (Méndez‐Fernández et al., 2024).

### 2.8 In vitro isometric tension recording

The soleus muscle was mounted by its tendons in a chamber for isometric tension recording. One tendon was connected to an optical transducer (World Precision Instruments, USA), while the other was fixed to the bottom of the chamber. Muscle length was adjusted to the optimal length for force generation (L_o_), at which maximal twitch force was obtained. Throughout the experiment, the muscle remained immersed in the mouse‐formulated physiological solution (described in Section 2.6) with continuous carbogen gas flow (95% O_2_, 5% CO_2_).

Within the recording chamber, two platinum–iridium electrodes were placed directly in the solution, avoiding direct contact with the muscle. These electrodes were connected to an isolated stimulation unit (Grass, USA) to induce fatigue, which was initiated 10 min after the muscle was mounted. The transducer was connected to an amplifier and an analog–digital interface (World Precision Instruments, USA), allowing acquisition of the tension generated by each muscle on a computer using MDAC software (World Precision Instruments, USA) (Gómez‐Barroso et al., 2020).

### 2.9 Fatigue protocol

Soleus muscle fatigue was induced through repetitive electrical stimulation. This was applied using electrical pulses of 100 V, 300 ms in duration and 45 Hz frequency, delivered via the isolated stimulator unit. The muscle was stimulated until its tension decreased by 60%–70% relative to the initial tension. The time to the muscle's fatigue threshold was recorded (Gómez‐Barroso et al., 2020).

### 2.10 Determination of oxidant production

The presence of oxidant species was determined using the fluorescence probe 2′,7'dichlorodihydrofluorescein diacetate (H_2_DCFDA) (D6883, Sigma‐Aldrich, St. Louis, MO, USA). 25 μg of protein from soleus muscle homogenates was placed in 2 mL of buffer containing 100 mM KCl, 10 mM HEPES, 3 mM KH_2_PO₄, and 3 mM MgCl_2_ (pH 7.4), and incubated with 12.5 μM H_2_DCFDA at 4 °C under constant agitation for 15 min. Basal fluorescence was then recorded after an equilibration period. Subsequently, the samples were agitated for 60 min to record the final fluorescence. Fluorescence changes were measured at excitation/emission wavelengths of 485 nm/520 nm using a spectrofluorophotometer (Shimadzu RF‐5301PC, Shimadzu, Kyoto, Japan). Results were expressed in arbitrary units (AU) per mg of protein (Bravo‐Sánchez et al., 2021). All measurements were performed in duplicate for each sample.

### 2.11 Catalase activity

Catalase activity was analyzed by measuring the conversion of H_2_O_2_ to O_2_ using a Clark‐type oxygen electrode connected to a biological oxygen monitor (5300A Biological Oxygen Monitor, YSI, Ohio, USA) (Peña‐Montes et al., 2019). First, 25 μg of protein from soleus muscle homogenates was resuspended in a 0.1 M phosphate buffer (pH 7.4) at 25°C and monitored for 1 min. Then, 6 mM H_2_O_2_ was added to the chamber, and the conversion of H_2_O_2_ to O_2_ was measured for 3 min using the oxygen electrode. Finally, 1.0 mM of sodium azide (S2002, Sigma‐Aldrich, St. Louis, MO, USA) was added to the chamber. O_2_ production was continuously monitored, and catalytic activity was expressed as μmol O_2_ produced per minute. Each measurement was performed in triplicate, and the final result was the mean slope of the curve used to quantify catalase activity in the sample. Results were expressed as μmol of H_2_O_2_ per mg of protein.

### 2.12 Determination of glutathione levels

Glutathione levels in the soleus muscle were determined using the method previously described by Rahman (Rahman et al., 2006), with some modifications. The total glutathione (GSH + GSSG) was determined at a protein concentration of 25 μg. Samples were deproteinized with a releasing solution (0.1% Triton X‐100, 0.6% sulfosalicylic acid, and 5 mM Na_2_EDTA in 50 mM potassium phosphate buffer, pH 7.5). This mixture underwent three sonication/ice cycles, each lasting 6 s. The samples were then subjected to two freeze–thaw cycles and centrifuged at 10,000 rpm for 5 min. The resulting supernatant (90 μL) was mixed with 50 mM potassium phosphate buffer, 0.1 mM 5,5′‐dithiobis (2‐nitrobenzoic acid) (DTNB) (D8130, Sigma‐Aldrich, St. Louis, MO, USA), and 0.1 units/mL glutathione reductase (G3564, Sigma‐Aldrich, St. Louis, MO, USA). The reaction was initiated by adding 50 μM β‐NADPH, and absorbance was measured for 5 min at 412 nm using a UV/Vis spectrophotometer (Shimadzu UV‐2550, Kyoto, Japan). The increase in absorbance at 412 nm was used to calculate the concentration of GSH + GSSG based on the molar extinction coefficient of glutathione. For the determination of oxidized glutathione (GSSG), samples were incubated for 1 h at room temperature with 0.2% 4‐vinylpyridine (V3204, Sigma‐Aldrich, St. Louis, MO, USA) to derivatize reduced glutathione (GSH). The samples were then processed according to the procedure described above to determine GSH + GSSG. The concentration of GSH was calculated by subtracting the GSSG concentration from the GSH + GSSG concentration.

### 2.13 Gene expression analysis by RT‐qPCR of *RYR1* , *SERCA1* , *NCX3* , and *Nrf2*

RNA was isolated from muscle tissue using the TRIzol™ (15596018, Invitrogen™, Thermo Fisher Scientific, Waltham, MA, USA) method (Thermo Fisher Scientific, Waltham, MA, USA). The RNA pellet was resuspended in 10 μL of nuclease‐free water. RNA quality was confirmed by 1% agarose gel electrophoresis, ensuring the presence of clear 28S and 18S rRNA bands. RNA concentration was determined spectrophotometrically using a Synergy HT reader (BioTek Instruments, Winooski, VT, USA).

One microgram of total RNA was used to synthesize complementary DNA (cDNA) using the High‐Capacity cDNA Reverse Transcription Kit (4368813, Applied Biosystems™, Thermo Fisher Scientific, Waltham, MA, USA). Gene expression was analyzed by RT‐qPCR using Brilliant III Ultra‐Fast SYBR® Green ROX QPCR master mix (600903, Agilent Technologies, Santa Clara, CA, USA) and the QuantStudio™ 3 thermocycler (Applied Biosystems™, Thermo Fisher Scientific, Waltham, MA, USA). Primer concentration for amplification was 300 nM. Amplification products were verified by analyzing the melting curve of each sample. Relative gene expression was calculated using the 2^−ΔΔCT^ method, with β‐actin as the endogenous reference gene. The PCR primer sequences used in this study are presented in Table 1.

**TABLE 1:** PCR primer sequences.

Gene	Primer sequences
*Nrf2*	Forward: 5′‐ACTACAGTCCCAGCAGGACAT‐3′ Reverse: 5′‐TCCTTCTGGAGTTGCTCTTG‐3′
*RyR1*	Forward: 5′‐CCAGTTTTTGCGGACGGATG‐3′ Reverse: 5′‐GTTCCAGAAAGCAAAGGCGG‐3′
*SERCA1*	Forward: 5′‐AACGCCTGCAATTCGGTGAT‐3′ Reverse: 5′‐GTGCCAACACGCACATAGTT‐3′
*NCX3*	Forward: 5′‐AGTCCTATGAGTTCAAGAGTACAG‐3′ Reverse: 5′‐AAGACCGTCAGGAAGTGCAT‐3′
*β‐actin*	Forward: 5′‐GAAAAGATGACCCAGATCATG‐3′ Reverse: 5′‐ATCACAATGCCAGTGGTAC‐3′

### 2.14 Statistical analysis

Before parametric analyses, data distribution was assessed using the Shapiro–Wilk normality test. Most datasets met the assumption of normality (*p* > 0.05). Homogeneity of variance and related ANOVA assumptions were evaluated within GraphPad Prism. When Prism indicated violations of these assumptions, appropriate corrections were applied.

Statistical comparisons were performed using a two‐way analysis of variance (ANOVA) with HFpEF (Control vs. HFpEF) and Exercise (Sedentary vs. Exercise) as independent factors. When a significant interaction or main effect was detected, Tukey's post hoc multiple comparisons test was applied. For datasets requiring correction, adjusted *p* values (Geisser–Greenhouse) were used as indicated by the software. Statistical significance was set at *p* < 0.05.

Data are presented as mean ± standard deviation (SD), with individual data points shown when appropriate. All statistical analyses were performed using GraphPad Prism software (version 8.0.1; GraphPad Software, La Jolla, CA, USA).

## 3 RESULTS

### 3.1 A high‐fat diet in combination with L‐NAME induces features consistent with an HFpEF‐like phenotype in mice: Effect of aerobic exercise

**FIGURE 2:**
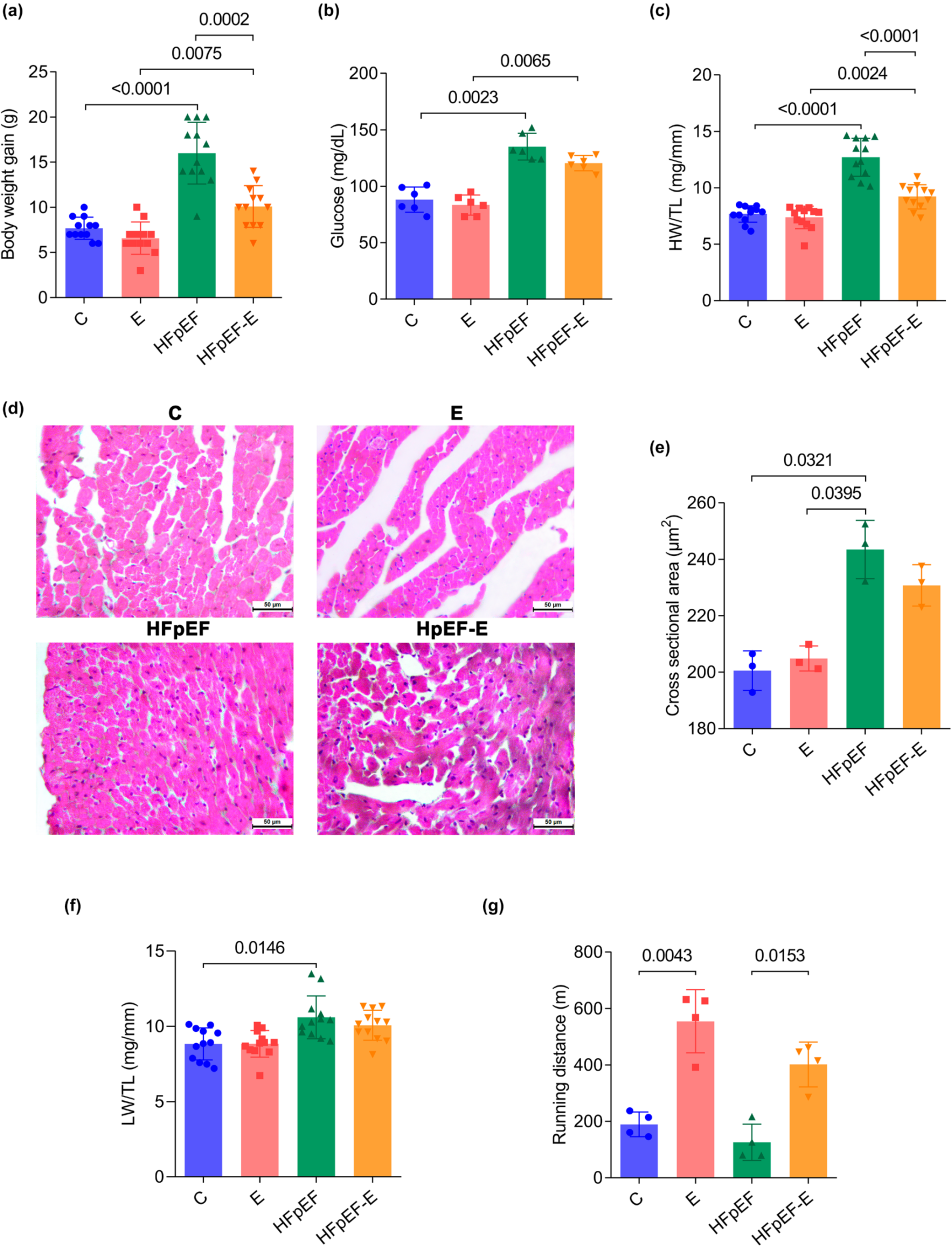
Eight weeks of a high‐fat diet combined with L‐NAME induce key clinical features of HFpEF in mice, and these effects are attenuated by aerobic exercise. (a) Body weight gain. (b) Glucose levels. (c) Ratio of heart weight to tibia length (HW/TL). (d) Representative micrographs of cardiac tissue cross‐sections stained with H&E. Magnification: 40×. Scale bar: 50 μm. (e) Cardiomyocyte cross‐sectional area derived from H&E micrographs. (f) Ratio of lung weight to tibia length (LW/TL). (g) Running distance during the exercise exhaustion test. For body weight gain, HW/TL and LW/TL *n* = 12 mice per group; for glucose *n* = 6; for cross‐sectional area *n* = 3; for running distance *n* = 4. Data are presented as mean ± SD. *p* < 0.05, two‐way ANOVA followed by Tukey's post hoc test. HFD, high‐fat diet; HW, heart weight; L‐NAME, Nω‐Nitro‐L‐arginine methyl ester; LW, lung weight; TL, tibia length.

After 8 weeks of treatment, the HFpEF group (HFD + L‐NAME‐treated mice) showed a significantly higher body weight (Figure 2a) compared with the C group (16 ± 3.4 g vs. 7.6 ± 1.2 g; *p* < 0.0001). The HFpEF‐E group exhibited a significantly lower body weight than the sedentary HFpEF group (10 ± 2.3 g; *p* = 0.0002), while no differences were observed between the C and E groups, suggesting an association between aerobic exercise and reduced body weight in HFpEF mice.

Blood glucose levels (Figure 2b) were significantly higher in the HFpEF group (135 ± 11.8 mg/dL) compared with the C group (88.1 ± 11.1 mg/dL; *p* < 0.0001). No significant differences were observed between HFpEF and HFpEF‐E groups, indicating that aerobic exercise did not normalize hyperglycemia under these conditions.

Heart weight normalized to tibia length (Figure 2c) was higher in the HFpEF group (12.6 ± 1.6 mg/mm; *p* < 0.0001) than in the C group (7.6 ± 0.7 mg/mm). The HFpEF‐E group presented a significantly lower heart weight compared with the sedentary HFpEF group (9.1 ± 1 mg/mm; *p* < 0.0001), while no differences were observed between the C and E groups. Histological analysis showed that the cardiomyocyte cross‐sectional area (Figure 2e) was significantly larger in the HFpEF group (243.4 ± 10.3 μm^2^) compared with the C group (200.5 ± 6.9 μm^2^; *p* = 0.0321). However, no significant differences were observed between the HFpEF and HFpEF‐E groups. Taken together, these findings confirm cardiac hypertrophy in the HFpEF model and suggest that exercise may partially attenuate its progression, although it does not fully reverse cellular hypertrophy.

Lung weight normalized to tibia length (Figure 2f) was higher in the HFpEF group (10.5 ± 1.4 mg/mm) compared with the C group (8.8 ± 1 mg/mm; *p* = 0.0146), with no significant differences between exercised groups and their respective controls, reflecting pulmonary congestion not modified by training.

Taken together, these findings indicate that HFD + L‐NAME‐treated mice exhibit cardiac hypertrophy, pulmonary congestion, and metabolic alterations, features consistent with an HFpEF‐like phenotype rather than direct confirmation of HFpEF.

In the exercise exhaustion test, the distance traveled (Figure 2g) by mice in the HFpEF group was lower, although without reaching statistical significance, compared with the C group. Exercised groups covered a significantly greater distance than their respective sedentary groups: E (554.6 ± 112.2 m; *p* = 0.0043) and HFpEF‐E (401.7 ± 79.7 m; *p* = 0.0153), demonstrating that aerobic training improves exercise tolerance in both healthy animals and the HFpEF model.

### 3.2 Effect of aerobic exercise on contraction strength and fatigue resistance time of the soleus muscle in the HFpEF model

**FIGURE 3:**
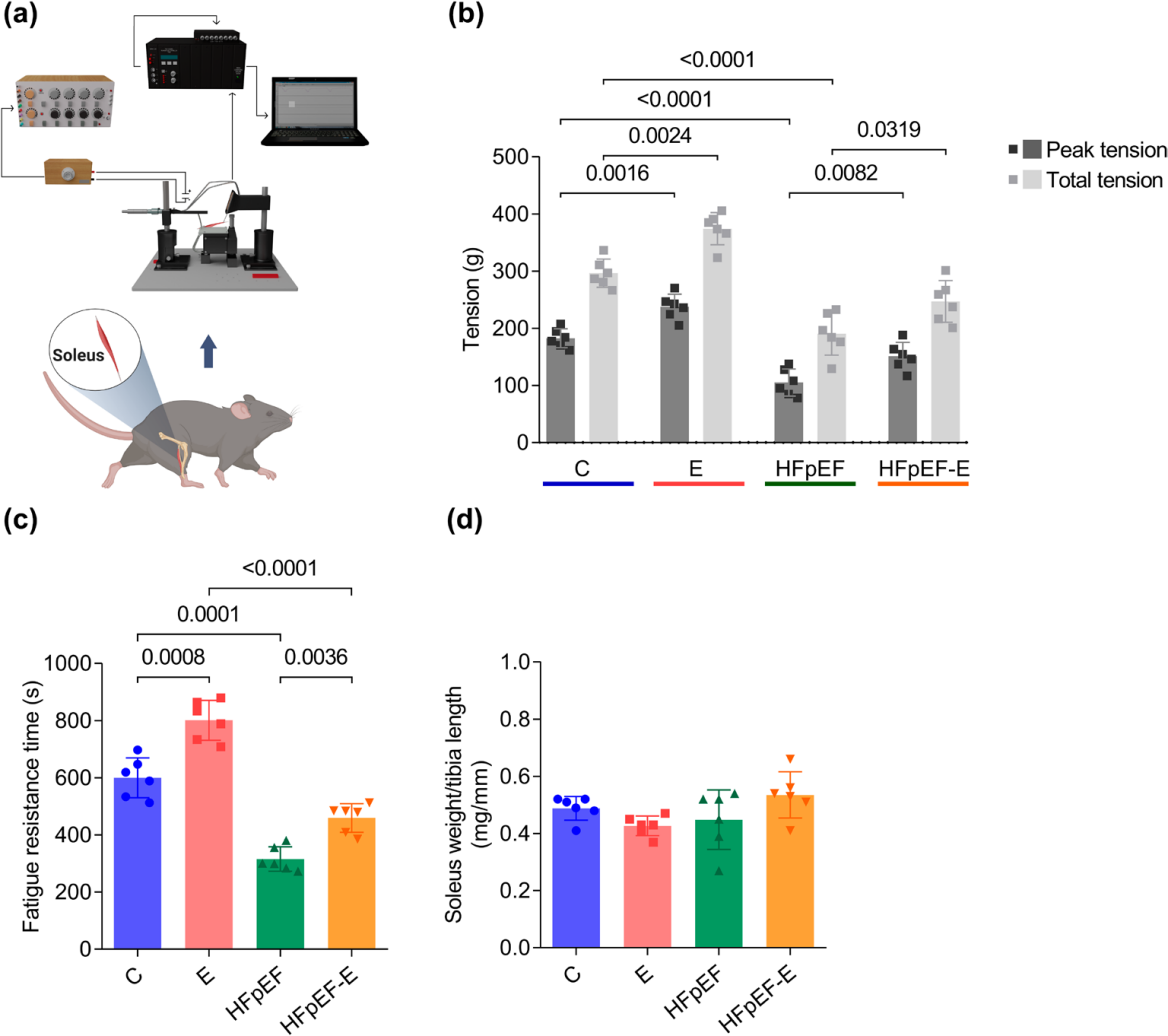
Effect of aerobic exercise on contraction force and fatigue resistance of the soleus muscle in the HFpEF model. (a) Schematic of the in vitro isometric tension recording used to evaluate muscle contraction force and fatigue resistance. Created with BioRender.com. (b) Maximum tension (dark gray bars) and total tension (light gray bars) of the soleus muscle. (c) Fatigue resistance time of the soleus muscle. (d) Ratio of soleus weight to tibial length. For tension, fatigue resistance time, and soleus weight/tibial length, *n* = 6 mice per group. Data are presented as mean ± SD. *p* < 0.05, two‐way ANOVA followed by Tukey's post hoc test.

Figure 3b shows the maximum tension and total tension of the soleus muscle. In the HFpEF group, the maximum tension was lower compared to the C group (105.2 ± 23.6 g vs. 182.6 ± 16.6 g; *p* < 0.0001), indicating a decrease in contractile function in this model. The E group showed a higher maximum tension compared to the C group (237.5 ± 22 g, *p* < 0.0016 vs. C), while the HFpEF‐E group exhibited a higher maximum tension compared to the sedentary HFpEF group (151.2 ± 24.3 g; *p* < 0.0082). Total tension followed a similar pattern. HFpEF showed lower total tension than the C group (190.8 ± 37.6 g vs. 296.4 ± 24.6 g; p < 0.0001). The E (374.2 ± 28.3 g; *p* = 0024 vs. C) and HFpEF‐E (246.9 ± 36.4 g; *p* < 0.0319 vs. HFpEF) groups showed higher values than their sedentary counterparts. Taken together, these findings indicate that aerobic exercise is associated with greater contractile capacity, even under pathological conditions such as the HFpEF model.

Figure 3c shows the fatigue resistance time of the soleus muscle. HFpEF exhibited a shorter fatigue resistance time than C (315.6 ± 42.9 s vs. 599.8 ± 69.3 s; *p* = 0.0001). The exercised groups showed higher values than their respective sedentary groups: HFpEF‐E (459.5 ± 49.9 s; *p* = 0.0036 vs. HFpEF) and E (801.3 ± 69.8 s; *p* = 0.0008 vs. C), indicating that aerobic exercise is associated with greater fatigue resistance.

Finally, no significant differences in soleus muscle weight normalized to tibia length (Figure 3d) were observed among the groups, suggesting that the observed changes are due to functional adaptations rather than morphological differences.

### 3.3 Aerobic exercise modulates oxidative stress markers in the soleus muscle of the HFpEF model

**FIGURE 4:**
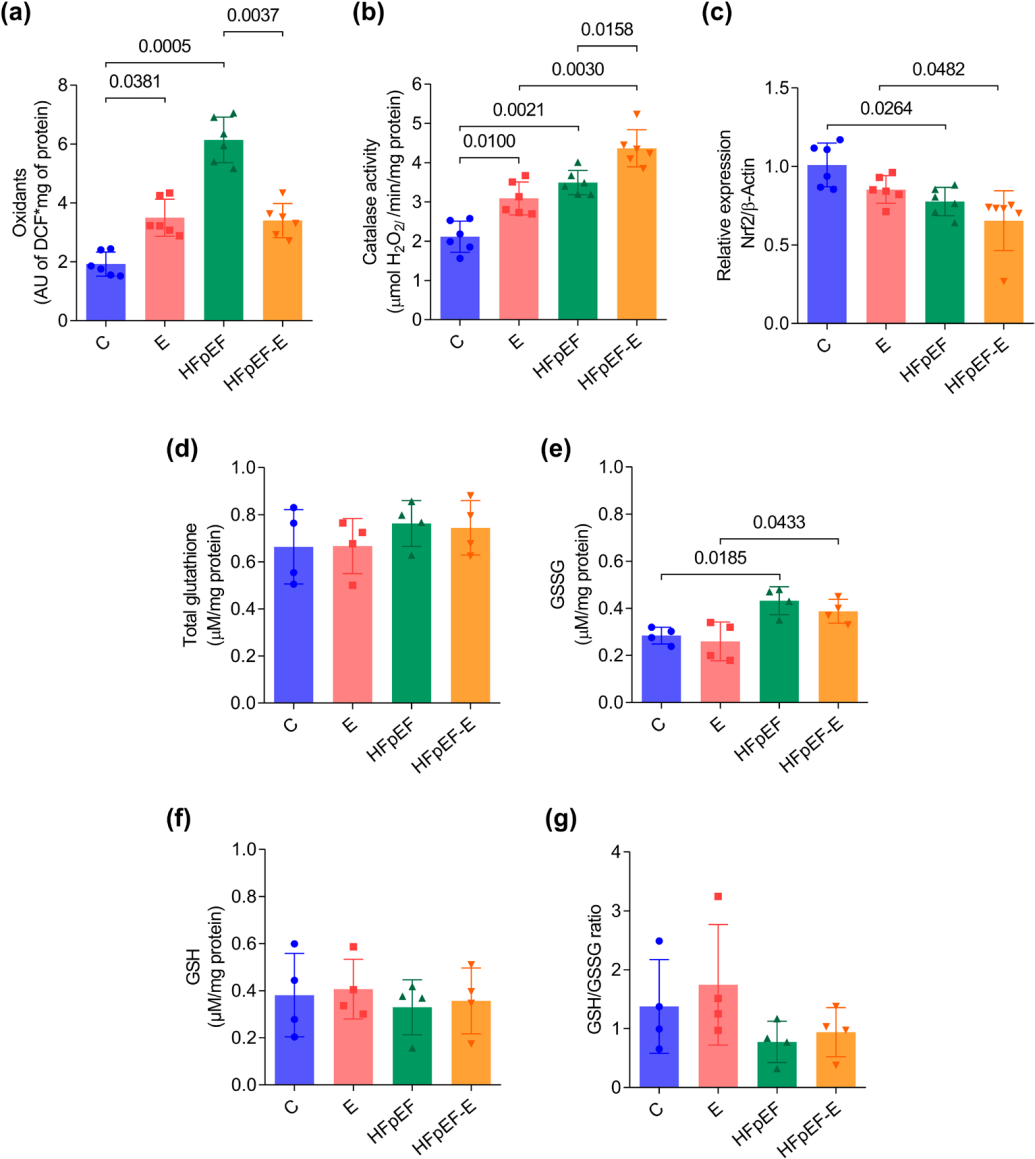
Effect of aerobic exercise on oxidative stress markers in the soleus muscle of the HFpEF model. (a) Total oxidants. (b) Catalase activity. (c) Nrf2. (d) Total glutathione (GSH + GSSG). (e) Oxidized glutathione (GSSG). (f) Reduced glutathione (GSH). (g) GSH/GSSG ratio. For oxidants, catalase activity, and Nrf2, *n* = 6 mice per group; for total glutathione, GSSG, GSH, and GSH/GSSG ratio, *n* = 4. Data are presented as mean ± SD. *p* < 0.05, two‐way ANOVA followed by Tukey's post hoc test. AU, arbitrary units; DCF, dichlorofluorescein; Nrf2, nuclear factor erythroid 2‐related factor 2.

Oxidant levels (Figure 4a) in the soleus muscle were significantly higher in the HFpEF group (6.1 ± 0.7 AU) compared to the C group (1.9 ± 0.4 AU; *p* = 0.0005). The HFpEF‐E group showed lower values than sedentary HFpEF (3.4 ± 0.5 AU; *p* = 0.0037). This indicates that, under HFpEF conditions, training is associated with reduced oxidant accumulation. Interestingly, the E group also showed higher values than C (3.4 ± 0.6 AU; *p* = 0.0381), although they remained well below those observed in HFpEF.

Catalase activity (Figure 4b) was higher in HFpEF (3.4 ± 0.3 μmol H_2_O_2_/min/mg prot; *p* = 0.0021) compared to C (2.1 ± 0.3 μmol H_2_O_2_/min/mg prot). The E group also showed higher values than C (3.0 ± 0.4 μmol H_2_O_2_/min/mg prot; *p* = 0.0100). Notably, HFpEF‐E exhibited the highest activity among all groups (4.3 ± 0.4 μmol H_2_O_2_/mg prot; *p* = 0.0158 vs. HFpEF). This finding suggests that catalase antioxidant activity is elevated in the soleus muscle of the HFpEF model and is further enhanced by exercise.

Given these changes, Nrf2 gene expression was assessed by RT‐qPCR, as this transcription factor is a key regulator of antioxidant defense. Nrf2 gene expression (Figure 4c) in the HFpEF group was lower compared to C (0.7 ± 0.09 vs. 1 ± 0.1; *p* = 0.0265). Thus, under the model conditions, HFpEF exhibits reduced Nrf2 levels compared to control, without evidence of exercise‐related differences.

No significant differences were observed among groups in GSH + GSSG levels (Figure 4d), GSH content (Figure 4f), or the GSH/GSSG ratio (Figure 4g). In contrast, GSSG levels (Figure 4e) were higher in HFpEF (0.4 ± 0.05 μM/mg prot; *p* = 0.0185) and HFpEF‐E (0.3 ± 0.05 μM/mg prot; *p* = 0.0433) compared to E (0.2 ± 0.08 μM/mg prot), with no significant differences between HFpEF and HFpEF‐E. These results suggest that the oxidative state associated with HFpEF persists within the glutathione system, even during exercise.

### 3.4 The soleus muscle in the HFpEF model showed lower mRNA expression of SERCA1 and NCX3, which was not restored by aerobic exercise

**FIGURE 5:**
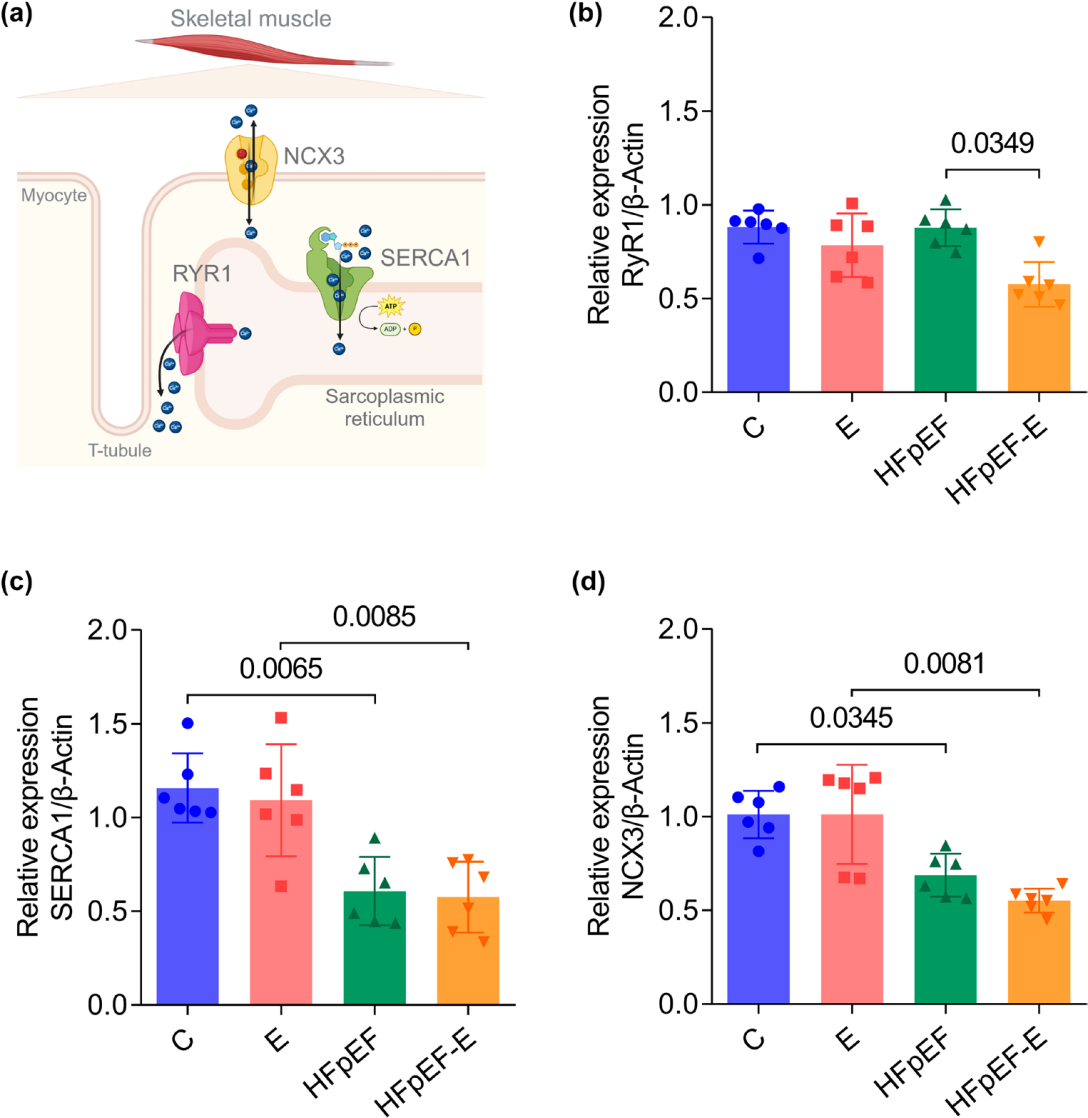
Effect of aerobic exercise on RyR1, SERCA1, and NCX3 gene expression in the soleus muscle of the HFpEF model. (a) Schematic representation of the function of genes involved in Ca^2+^ release and reuptake in the soleus muscle. RyR1, Ca^2+^ release channel in the sarcoplasmic reticulum; SERCA1 transports Ca^2+^ from the myocyte cytoplasm back into the sarcoplasmic reticulum; NCX3 regulates intracellular Ca^2+^ levels via Na^+^/Ca^2+^ exchange across the cell membrane. Created with BioRender.com. (b) RyR1. (c) SERCA1. (d) NCX3. For RyR1, SERCA1, and NCX3, *n* = 6 mice per group. Data are presented as mean ± SD. *p* < 0.05, two‐way ANOVA followed by Tukey's post hoc test. NCX3, sodium/calcium exchanger type 3; RyR1, ryanodine receptor type 1; SERCA1, sarcoplasmic reticulum Ca^2+^‐ATPase 1.

Gene expression of Ca^2+^‐handling related genes in the soleus muscle of the HFpEF model was analyzed by RT‐qPCR. No significant differences were observed in RyR1 expression (Figure 5b) between HFpEF and C groups. However, RyR1 expression values were lower in the HFpEF‐E group (0.5 ± 0.1; *p* = 0.0349) compared with sedentary HFpEF (0.8 ± 0.09).

SERCA1 expression (Figure 5c) was lower in the HFpEF (0.6 ± 0.1; *p* < 0.0017) and HFpEF‐E (0.5 ± 0.1; *p* = 0.0065) groups compared with the C (1.1 ± 0.1) and E (1 ± 0.2) groups. Aerobic exercise did not significantly alter SERCA1 expression in the E and HFpEF‐E groups compared with the sedentary groups.

Similarly, NCX3 expression (Figure 5d) was lower in the HFpEF (0.6 ± 0.1; p = 0.0065) compared with C (1 ± 0.1). No significant differences were observed between the exercised and sedentary groups. In the HFpEF model, the genes encoding Ca^2+^‐handling proteins showed reduced expression (SERCA1 and NCX3), and aerobic exercise did not significantly modify these levels, whereas RyR1 showed lower expression only in HFpEF‐E.

## 4 DISCUSSION

Aerobic training has been established as a non‐pharmacological strategy for HFpEF due to its anti‐inflammatory and antioxidant effects (Margaritelis et al., 2020). Although improvements in cardiac function and quality of life have been documented (Crisci et al., 2022; Dieberg et al., 2015), its impact on skeletal muscle function remains incompletely understood. Muscle dysfunction contributes to exercise intolerance, a key prognostic factor in HFpEF (Gupte & Hamilton, 2016; Saw et al., 2021), and it has been suggested that alterations in Ca^2+^ homeostasis and antioxidant capacity may be involved (Bolaños & Calderón, 2022; Michelucci et al., 2021; Qaisar et al., 2018).

The present study provides novel evidence that aerobic exercise improves skeletal muscle function in a model with features consistent with HFpEF, primarily through modulation of redox homeostasis rather than normalization of Ca^2+^‐handling gene expression.

Preclinical studies in murine models show that aerobic exercise can improve muscle function by altering redox homeostasis and altering the expression of proteins involved in Ca^2+^ handling. In a model of heart failure induced by sympathetic hyperactivity, aerobic training increased the expression of sarcoplasmic proteins involved in Ca^2+^ release and reuptake (DHPR, RyR, SERCA, and NCX) in soleus and plantaris, accompanied by improvements in exercise tolerance and muscle function (Bueno Jr et al., 2010). Similar findings were observed in trained healthy mice, with increases in DHPR, RyR, and SERCA and an improved redox state (Ferreira et al., 2010).

However, it is not known whether these effects translate to the HFpEF context, which is characterized by a complex, multisystemic pathology (Gevaert et al., 2022). In this context, our findings are consistent with the notion that aerobic exercise modulates skeletal muscle function and redox balance in a murine model with features consistent with HFpEF, specifically affecting contractility, fatigue resistance, and oxidative stress parameters in the soleus muscle. The soleus was selected due to its predominance of oxidative type I fibers, which consume a large proportion of oxygen during physical activity and require efficient Ca^2+^ handling and redox balance (Saw et al., 2022), making it a suitable muscle to evaluate exercise‐induced adaptations in HFpEF.

### 4.1 Animal model

Our results are consistent with previous reports, which confirm that the combination of HFD and L‐NAME produces features consistent with HFpEF described in the literature, rather than directly confirming HFpEF in our experimental cohort. After 8 weeks, mice exhibited increased body weight and elevated fasting glucose levels, in agreement with previous studies linking HFD to higher fat mass and insulin resistance (Thomas et al., 2014; Vargas‐Robles et al., 2015). In this context, HFD has been reported to induce chronic inflammation and intramyocellular lipid accumulation, which impair insulin‐GLUT4 signaling (Eshima, 2021; Wood et al., 2021). Although aerobic exercise reduced body weight in the HFpEF‐E group in our study, it did not normalize glucose levels. This suggests that weight reduction alone during the applied training protocol may be insufficient to reverse hyperglycemia in this model. Notably, insulin sensitivity was not directly assessed in the present study; however, these findings are consistent with evidence indicating that alterations in fat distribution and skeletal muscle metabolic capacity play a more determinant role in glucose homeostasis (Oliveros et al., 2014; Thyfault & Bergouignan, 2020).

Cardiac hypertrophy was evident in the HFpEF group (HFD + L‐NAME‐treated mice), reflected by increased heart weight and cardiomyocyte cross‐sectional area. These findings align with previous descriptions of cardiac remodeling models with features associated with HFpEF, in which mechanisms such as alterations in the NO–cGMP–PKG pathway, oxidative stress, and inflammation have been implicated (Méndez‐Fernández et al., 2024; Paulus & Tschöpe, 2013). In our study, aerobic exercise partially attenuated these structural alterations. Although the underlying mechanisms were not directly evaluated, this attenuation is consistent with previous evidence indicating that aerobic training can modulate the early stages of cardiac remodeling in cardiovascular diseases, including HFpEF (Subbotina et al., 2015; Szaroszyk et al., 2022; Teles et al., 2023).

Interestingly, although heart weight was reduced in the exercised group, cardiomyocyte cross‐sectional area remained unchanged. This apparent discrepancy may be explained by the contribution of non‐myocyte components to total heart mass, including extracellular matrix remodeling, interstitial fluid content, and inflammatory processes, which may be modulated by exercise independently of cardiomyocyte size (Paulus & Tschöpe, 2013).

Lung weight, an indirect marker of pulmonary congestion, was elevated in the HFD + L‐NAME‐treated group, consistent with previous reports in this experimental model (Schiattarella et al., 2019). This finding has been described as potentially related to increased ventricular filling pressure and consequent pulmonary venous hypertension (Papps & Filippatos, 2011; Reddy et al., 2019). In our study, exercise did not modify this parameter, suggesting that the duration and/or intensity of the training protocol was insufficient to reverse pulmonary congestion‐associated structural changes in this experimental setting.

Finally, although the distance covered in the exercise exhaustion test was lower in the HFpEF group, the difference did not reach statistical significance, which contrasts with previous reports. This discrepancy may be partially explained by the limited sample size in the exercise test (*n* = 4), which reduces statistical power to detect group differences. Nevertheless, the trained animals showed improved performance, consistent with studies demonstrating that aerobic exercise enhances functional capacity in HFpEF (Leggio et al., 2020; Quiriarte et al., 2024).

### 4.2 Skeletal muscle

In our study, HFpEF mice showed reduced contraction capacity and fatigue resistance in the soleus muscle, consistent with preclinical models of muscle dysfunction in HFpEF (Bowen et al., 2015; Kelley et al., 2022; Quiriarte et al., 2024). In a Dahl salt‐sensitive rat model, fiber atrophy, together with mitochondrial and functional alterations, was described in the soleus (Bowen et al., 2015). Likewise, in a preclinical postmenopausal HFpEF model, reduced skeletal muscle strength, impaired mitochondrial respiration, and redox imbalance were observed, reinforcing the role of peripheral skeletal muscle dysfunction in this pathology (Kelley et al., 2022). In line with these studies, using the same HFpEF mouse model employed in our work, it has been demonstrated that exercise intolerance is associated with incomplete oxidation of lipids and branched‐chain amino acids in skeletal muscle (Quiriarte et al., 2024). It is worth noting that an essential feature of HFpEF is the coexistence of metabolic comorbidities such as obesity, type 2 diabetes mellitus, and hypertension, which favor the establishment and progression of the syndrome (Bozkurt et al., 2021; Wood et al., 2021). In this context, it has been reported that in obese rats, contraction and fatigue resistance of the soleus muscle are reduced, associated with fat infiltration, fiber‐type shifts, metabolic alterations, and increased oxidative stress (Gómez‐Barroso et al., 2022). Accordingly, our findings of contractile dysfunction in the soleus add to this body of evidence, showing that, in addition to metabolic alterations, intrinsic functional deficits in skeletal muscle likely contribute to exercise intolerance in HFpEF.

In the HFpEF‐E group, both contractile capacity and fatigue resistance were significantly improved with aerobic exercise. These findings are consistent with reports from preclinical models showing that physical training attenuated mitochondrial and functional alterations in skeletal muscle (Bowen et al., 2015). Similarly, it has been demonstrated that in the HFpEF mouse model, voluntary running restored lipid and branched‐chain amino acid oxidation, thereby improving exercise capacity, reinforcing the role of muscle metabolism as a determinant of this pathology (Quiriarte et al., 2024). In our study, the observed improvement could be related to enhanced metabolic efficiency, reduced oxidative stress, and decreased fat infiltration, mechanisms linked to improved contractile capacity and fatigue resistance (Gallagher et al., 2023; Gómez‐Barroso et al., 2022; Martinez‐Huenchullan et al., 2018).

Furthermore, no differences in soleus muscle weight were observed between groups, indicating that functional dysfunction occurs in the absence of overt muscle atrophy. In the literature, it has been noted that contractile impairment may be associated with metabolic, inflammatory, or structural alterations in muscle fibers rather than with loss of mass (Gallagher et al., 2023). In this context, the reduced contractile capacity and fatigue resistance observed may be linked to alterations in excitation–contraction coupling (ECC), including potential changes in molecular components associated with Ca^2+^ regulation.

Although the present study evaluated only the expression of ECC‐related genes (RyR1, SERCA1, and NCX3) and did not directly assess intracellular Ca^2+^ dynamics, the results suggest that skeletal muscle functional deficits in HFpEF may be associated with alterations in the contractile machinery, rather than providing direct evidence of altered Ca^2+^ handling. Importantly, mRNA expression does not necessarily reflect protein abundance or functional activity of Ca^2+^‐handling components, and even protein levels may not directly correlate with channel or pump function due to post‐translational modifications.

Regarding the redox state of the soleus muscle, the HFpEF group showed higher oxidant levels, suggesting that an excess of oxidants may impair muscle contractility and promote fatigue. This finding is consistent with previous reports in models of obesity, type 2 diabetes mellitus, and hypertension, where increased oxidants in skeletal muscle are associated with contractile dysfunction (Bravo Sánchez et al., 2024; Gómez‐Barroso et al., 2022; Sánchez‐Duarte et al., 2022). Previous studies have proposed interactions between Ca^2+^ homeostasis and redox balance in skeletal muscle; however, these mechanisms were not directly evaluated in the present study. Therefore, the contribution of Ca^2+^‐dependent pathways to oxidant production should be interpreted cautiously in this context, particularly given that Ca^2+^‐handling was not directly assessed at the functional or protein level in this study (Allen et al., 2008; Bouviere et al., 2021; Powers & Jackson, 2008). In contrast, in the E group, soleus contractility improved, possibly due to a moderate and controlled increase in oxidant levels, since suboptimal levels induced by exercise can activate adaptive signaling pathways involved in mitochondrial biogenesis and antioxidant defense (Margaritelis et al., 2020). This finding indicates that increased oxidant levels do not necessarily impair muscle function and may instead reflect a physiological adaptive response to exercise. In the HFpEF‐E group, soleus contractility improved, accompanied by a reduction in oxidant levels, compared with the sedentary HFpEF group, suggesting that aerobic exercise contributes to restoring redox balance in skeletal muscle, likely through the activation of endogenous antioxidant systems (Bravo Sánchez et al., 2024; Gómez‐Barroso et al., 2022). This protective effect could help preserve mitochondrial integrity and improve muscle performance under HFpEF conditions, consistent with studies highlighting the therapeutic value of exercise in modulating oxidative stress in chronic diseases (Gallagher et al., 2023; Margaritelis et al., 2020).

Catalase activity was higher in the HFpEF group, despite lower Nrf2 mRNA expression, suggesting a compensatory antioxidant response mediated by alternative regulatory pathways, such as FoxO3, a positive transcriptional regulator of catalase (Kelley et al., 2022; Klotz et al., 2015; Pang et al., 2015). A chronic inflammation–mediated suppression of Nrf2, possibly via NF‐κB activation, could explain this discrepancy, as the imbalance between Nrf2 and NF‐κB pathways has been associated with various pathologies (Gao et al., 2021; Wardyn et al., 2015). In the trained groups, catalase activity was also higher without changes in Nrf2 mRNA expression, suggesting that exercise‐induced antioxidant responses may be mediated through Nrf2‐independent mechanisms and/or post‐transcriptional regulation. These findings should be interpreted cautiously, as the underlying regulatory mechanisms were not directly assessed in the present study. In this context, FoxO3 has been identified as a key regulator of muscle adaptations to exercise, promoting the expression of antioxidant enzymes such as catalase in response to intracellular oxidative stress (Kim et al., 2022; Sanchez et al., 2014). Additionally, the lack of change in Nrf2 expression may reflect limited sensitivity to detect transcriptional differences, rather than the absence of functional activation. These findings support the idea that antioxidant regulation in skeletal muscle involves multiple, interconnected signaling pathways that may vary depending on disease stage and stimulus type.

Regarding glutathione, the HFpEF group showed higher GSSG levels, with no significant changes in GSH + GSSG, GSH, or the GSH/GSSG ratio. These findings suggest a shift toward a more oxidized glutathione state, which may contribute to muscle dysfunction in this model, consistent with previous reports showing that increased GSSG was associated with decreased contractility and fatigue resistance in skeletal muscle from obesity and hypertension models (Bravo Sánchez et al., 2024; Gómez‐Barroso et al., 2022). However, glutathione‐related measurements showed considerable variability and limited group differences; therefore, these results should be interpreted cautiously and considered complementary to the oxidant and catalase activity data rather than as definitive evidence of redox restoration. On the other hand, aerobic exercise did not significantly alter glutathione levels in the trained groups. Previous studies have reported that GSH content and the GSH/GSSG ratio can increase with exercise, while GSSG decreases; these apparent discrepancies likely reflect differences in exercise protocols, duration, intensity, and the experimental models employed, as well as differences in statistical power associated with sample size (Elokda & Nielsen, 2007; Ferreira et al., 2010; Liu et al., 2000).

Intracellular Ca^2+^ regulation is essential for skeletal muscle contractility, and RyR1, SERCA1, and NCX3 participate in Ca^2+^ release, reuptake, and extrusion during excitation–contraction coupling (Bolaños & Calderón, 2022; Michelucci et al., 2021). Therefore, alterations in the expression of these genes may be associated with skeletal muscle dysfunction in HFpEF.

In our study, the HFpEF group showed lower mRNA expression of SERCA1 and NCX3, while RyR1 remained unchanged. These findings suggest potential alterations in the expression of molecular components associated with Ca^2+^ regulation, rather than direct evidence of altered Ca^2+^ regulation, which may contribute to muscle dysfunction and fatigue in HFpEF (Bueno Jr et al., 2010; Eshima, 2021; Méndez‐Fernández et al., 2024; Munkvik et al., 2010). Previous studies have proposed that alterations in SERCA1 and NCX expression may influence Ca^2+^ homeostasis and contribute to skeletal muscle dysfunction (Allen et al., 2008; Ferreira et al., 2010; Gallagher et al., 2023; Michelucci et al., 2021). However, in the absence of direct measurements of intracellular Ca^2+^ dynamics, these mechanisms should be interpreted as plausible hypotheses rather than definitive conclusions.

In the E group, exercise did not alter RyR1, SERCA1, or NCX3 mRNA expression, unlike animal studies, which show that exercise increases the expression and activity of these proteins in healthy groups (Bueno Jr et al., 2010; Ferreira et al., 2010). In the HFpEF‐E group, lower RyR1 mRNA expression was observed, without significant changes in SERCA1 or NCX3 compared with the sedentary HFpEF group. These findings suggest that improvements in muscle function may occur independently of changes in the expression of Ca^2+^‐handling‐related genes evaluated in this study. In contrast, Bueno et al. (Bueno Jr et al., 2010) reported that exercise restored these protein levels to values similar to those of controls in a murine model of heart failure induced by sympathetic hyperactivity, characterized by significant systolic dysfunction. This phenotype corresponds to heart failure with reduced ejection fraction (HFrEF); therefore, these discrepancies may be attributed to differences in both the exercise protocol and the underlying heart failure phenotype, since HFrEF and HFpEF present distinct pathophysiological substrates (Reiken et al., 2003). The lower RyR1 levels in HFpEF‐E are consistent with Munkvik et al. (2010), who reported reduced RyR content in the trained leg of patients with chronic heart failure, without concomitant alterations in Ca^2+^ leak or release kinetics, suggesting that reduced RyR expression does not necessarily translate into impaired Ca^2+^ release.

### 4.3 Study limitations

This study has several limitations that should be considered when interpreting the results. First, the exercise protocol lasted 4 weeks, which may be insufficient to induce sustained skeletal muscle adaptations in a chronic condition such as HFpEF; therefore, longer intervention studies are required to assess the persistence and magnitude of exercise‐induced adaptations. Second, the molecular analysis was limited to three calcium‐regulating genes assessed by RT‐qPCR, without evaluation of corresponding protein expression (RyR1, SERCA1, and NCX3) or direct measurements of intracellular Ca^2+^, thereby restricting the functional interpretation of calcium regulation in this model.

Additionally, only male C57BL/6J mice were included. Although this limits the direct extrapolation of the findings to females, who represent a large proportion of HFpEF patients, recent reports indicate that approximately 45% of patients are male (Lam et al., 2019) and that no significant sex‐related differences in skeletal muscle characteristics have been consistently observed (Espino‐Gonzalez et al., 2024). Nevertheless, future studies should include female animals and aging models, as both sex and age are strongly associated with HFpEF pathophysiology in the clinical setting.

Another important limitation is the variability in the number of animals across different experiments. The reduced sample size in some analyses was primarily due to the small size of the soleus muscle, which limited protein yield for biochemical assays; sample loss during tissue handling; the technical complexity of ex vivo tension recordings; and the need to repeat certain assays. Accordingly, the sample size for each analysis was determined by tissue availability and successful completion of the corresponding experimental procedure. All muscles that yielded sufficient material to generate reliable measurements were included; however, the limited sample size reduces statistical power and warrants cautious interpretation of the results, which should be considered preliminary. This is particularly relevant for glutathione‐related measurements, which showed greater variability and limited group differences.

Finally, although the murine model used—HFD + L‐NAME feeding—has been previously reported to produce features consistent with HFpEF (Schiattarella et al., 2019), direct cardiac functional assessments, such as echocardiographic measurements of LVEF or diastolic function (e.g., E/E′ ratio), were not performed in the evaluated cohort. Therefore, the presence of HFpEF in our experimental animals cannot be definitively confirmed.

Accordingly, the findings of this study should be interpreted within the context of a model exhibiting features consistent with an HFpEF‐like phenotype, as supported by cardiac hypertrophy, pulmonary congestion, and metabolic alterations observed in our experimental groups.

Therefore, extrapolation of these findings to human HFpEF should be made with caution. Consequently, the results should be interpreted prudently and confirmed in future studies with larger cohorts and greater biological diversity in terms of sex and age. Despite these limitations, the present study provides novel insight into the effects of aerobic exercise on skeletal muscle function and redox homeostasis in a model with features consistent with HFpEF.

## 5 CONCLUSION

In summary, our study demonstrates that aerobic exercise improves contractile function, fatigue resistance, and redox balance in the soleus muscle of a murine model with features consistent with HFpEF. These benefits appear to occur predominantly through metabolic and antioxidant adaptations, rather than through changes in the expression of the evaluated Ca^2+^‐regulating genes. Despite limitations, including a relatively short intervention duration, a limited sample size, and a restriction to male animals, our findings provide novel preclinical evidence supporting aerobic exercise as a strategy to mitigate skeletal muscle dysfunction associated with conditions consistent with HFpEF. Future studies incorporating longer training protocols, larger and more diverse experimental cohorts, and integrated molecular, protein‐level, and functional analyses are warranted further to elucidate the mechanisms underlying exercise‐induced muscle adaptations in HFpEF.

## AUTHOR CONTRIBUTIONS

**Cielo Martínez Martínez:** Conceptualization; data curation; formal analysis; investigation; methodology; resources; validation; visualization. **Jorge Fragoso Medina:** Data curation; formal analysis; investigation; methodology; project administration; supervision; validation. **Estefanía Bravo Sánchez:** Data curation; formal analysis; methodology; validation; visualization. **Bianca Nieblas:** Data curation; investigation; methodology; supervision. **Alfredo Saavedra Molina:** Data curation; project administration; resources; supervision. **Christian Cortés Rojo:** Data curation; project administration; resources; supervision. **Salvador Manzo Ávalos:** Data curation; project administration; resources; supervision. **Noemí García:** Conceptualization; funding acquisition; investigation; methodology; project administration; resources; supervision. **Rocío Montoya Pérez:** Conceptualization; funding acquisition; investigation; methodology; project administration; resources; supervision.

## CONFLICT OF INTEREST STATEMENT

The authors have no conflict of interest to declare.

## ETHICS STATEMENT

The animal study protocol was approved by the Ethics and Biosafety Committee of the Institute of Chemical‐Biological Research, Michoacan University of Saint Nicholas of Hidalgo (Ref. No. 06/2022, approval date 12‐01‐2022). The committee guaranteed the use of mice (Mus musculus) of the C57Bl/6J strain following the general regulations of the Official Mexican Standard for the use and maintenance of laboratory animals (NOM‐062‐ZOO‐1999).

## Data Availability

All data and materials used in the analysis are available in some form to any researcher for purposes of reproducing or extending the analysis.
